# Prevalence of potentially traumatic events and symptoms of depression, anxiety, hazardous alcohol use, and post-traumatic stress disorder among people with HIV initiating HIV care in Cameroon

**DOI:** 10.1186/s12888-023-04630-1

**Published:** 2023-03-09

**Authors:** Angela M. Parcesepe, Lindsey M. Filiatreau, Peter Vanes Ebasone, Anastase Dzudie, Brian W. Pence, Milton Wainberg, Marcel Yotebieng, Kathryn Anastos, Eric Pefura-Yone, Denis Nsame, Rogers Ajeh, Denis Nash

**Affiliations:** 1 Gillings School of Global Public Health, Department of Maternal and Child Health, University of North Carolina at Chapel Hill, Chapel Hill, NC USA; 2 University of North Carolina at Chapel Hill, Carolina Population Center, Chapel Hill, NC USA; 3 Department of Psychiatry, School of Medicine, Washington University in St. Louis, St. Louis, MO USA; 4 Clinical Research Education Networking and Consultancy, Yaounde, Cameroon; 5 Department of Psychiatry, Columbia University and New York State Psychiatric Institute, New York, NY USA; 6 Albert Einstein College of Medicine, Department of Medicine, Bronx, NY USA; 7 Albert Einstein College of Medicine, Departments of Medicine and Epidemiology & Population Health, Bronx, NY USA; 8 Jamot Hospital, Yaounde, Cameroon; 9 Bamenda Regional Hospital, Bamenda, Cameroon; 10 Institute for Implementation Science in Population Health, City University of New York, New York, NY USA

**Keywords:** Trauma, Mental health, HIV, Cameroon

## Abstract

**Background:**

This study explored the relationship between specific types of potentially traumatic events (PTEs) and symptoms of mental health disorders among people with HIV (PWH) in Cameroon.

**Methods:**

We conducted a cross-sectional study with 426 PWH in Cameroon between 2019–2020. Multivariable log binominal regression was used to estimate the association between exposure (yes/no) to six distinct types of PTE and symptoms of depression (Patient Health Questionnaire-9 score > 9), PTSD (PTSD Checklist for DSM-5 score > 30), anxiety (Generalized Anxiety Disorder-7 scale score > 9), and hazardous alcohol use (Alcohol Use Disorders Identification Test score > 7 for men; > 6 for women).

**Results:**

A majority of study participants (96%) reported exposure to at least one PTE, with a median of 4 PTEs (interquartile range: 2–5). The most commonly reported PTEs were seeing someone seriously injured or killed (45%), family members hitting or harming one another as a child (43%), physical assault or abuse from an intimate partner (42%) and witnessing physical assault or abuse (41%). In multivariable analyses, the prevalence of PTSD symptoms was significantly higher among those who reported experiencing PTEs during childhood, violent PTEs during adulthood, and the death of a child. The prevalence of anxiety symptoms was significantly higher among those who reported experiencing both PTEs during childhood and violent PTEs during adulthood. No significant positive associations were observed between specific PTEs explored and symptoms of depression or hazardous alcohol use after adjustment.

**Conclusions:**

PTEs were common among this sample of PWH in Cameroon and associated with PTSD and anxiety symptoms. Research is needed to foster primary prevention of PTEs and to address the mental health sequelae of PTEs among PWH.

## Introduction

Exposure to potentially traumatic events (PTE) is common among people with HIV (PWH) and more common among PWH compared to the general population [1–4]. PTEs can take many forms, occur throughout the life course, and include violent and non-violent events in which an individual is exposed to actual or threatened death, serious injury, or sexual violence [5]. In high-resource settings, it has been estimated that 30% of PWH have experienced childhood physical or sexual abuse and 68–95% of women with HIV have experienced intimate partner violence (IPV) [6–8]. Research on exposure to PTEs among PWH in sub-Saharan Africa (SSA) remains limited. However, a meta-analysis estimated that 33% of women with HIV in SSA have experienced IPV [9]. Other studies have reported substantially higher prevalence of IPV among women with HIV across global settings [10, 11]. For example, a study of women with HIV in South Africa found that 81% of respondents reported having experienced physical or sexual IPV in their lifetime [11]. Research with pregnant women with HIV in Cameroon found that more than half (63%) reported having experienced IPV in the past 12 months [10]. While research remains scare, experiences of childhood abuse and maltreatment have been commonly reported among PWH in sub-Saharan Africa [1, 12]. A study of PWH in Tanzania found that 53% of PWH reported physical neglect in childhood while a study of women with HIV in South Africa found that 11% of respondents reported childhood sexual abuse [12]. In addition, existing research has shown that having experienced multiple PTEs is common among PWH, with a study with women with HIV in South Africa reporting that almost half (46%) of respondents reported more than one PTE [11]. A study with PWH in Tanzania found that participants reported an average of three lifetime PTEs [12].

PTEs, including those experienced early in life, can have lasting physical and mental health effects [13–15]. Among PWH, traumatic events can increase risk of mental health and substance use disorders such as depression, anxiety, alcohol use disorder, and post-traumatic stress disorder (PTSD), and may contribute to poor HIV treatment outcomes, including delayed HIV diagnosis, delayed linkage to HIV care, suboptimal adherence to antiretroviral therapy (ART), unsuppressed viral load, and virologic failure [1, 4, 16–18]. Given the existing stressors of managing a chronic illness and limited availability of formal mental health care in many regions of SSA, PTEs may be particularly detrimental to the physical and mental well-being of PWH in SSA [19].

HIV continues to be a major public health problem throughout SSA, including in Cameroon, one of the largest countries in Central Africa. There are approximately 500,000 PWH in Cameroon. Despite recent progress, Cameroon remains far from achieving the UNAIDS’ goals of having 95% of PWH know their status, 95% of PWH who know their status on ART, and 95% of PWH on ART virally suppressed. Overall, 78% of PWH in Cameroon know their status and 74% of PWH in Cameroon are receiving ART [20]. Greater understanding of the prevalence of lifetime exposure to PTEs and the relationship between PTE exposure and mental health can inform strategies to improve the mental health and HIV treatment outcomes of PWH who have experienced trauma. Studies that investigate the prevalence and impact of PTEs by type are needed in SSA where the burden of HIV is great and research into the prevalence and impact of trauma remains limited. The aims of this study are to estimate the prevalence and type of lifetime PTEs experienced among PWH initiating HIV care in Cameroon and the relationship between types of PTEs and symptoms of depression, anxiety, PTSD, and hazardous alcohol use.

## Methods

### Data collection

This cross-sectional study has been previously described [21]. Briefly, data were collected from interviews with 426 individuals initiating HIV care at three HIV treatment clinics in Cameroon between June 2019 and March 2020. Individuals were eligible to participate if they were 21 years or older and enrolling in HIV care at one of the study clinics. Individuals who met the eligibility criteria were invited to participate. Data collection included questions on mental health, substance use, psychosocial stressors, PTEs, and sociodemographics. This study was approved by the Institutional Review Board at the University of North Carolina at Chapel Hill and the National Ethical Committee of Research for Human Health in Cameroon. All participants provided written informed consent.

### Measures

#### Lifetime traumatic events

We used a modified version of the Life Events Checklist for DSM-5 to measure exposure to twelve specific, contextually relevant lifetime PTEs. Events of interest included: seeing people hitting or harming one another in your family as a child, physical assault or abuse as a child, sexual assault or rape as a child, physical assault or abuse as an adult from an intimate partner, physical assault or abuse in adulthood from someone other than an intimate partner, sexual assault or rape in adulthood, seeing someone physically assaulted or abused, seeing someone seriously injured or killed, experiencing a natural disaster, experiencing a serious accident or fire, exposure to war, and losing a child through death. One additional question asked individuals to identify any other traumatizing event experienced during childhood or adulthood. We explored the free response text describing any “other” type of experienced lifetime traumatic events and recategorized these events if they fit more appropriately in a pre-defined category (e.g., one individual who reported death of a child through childbirth had not reported loss of a child to death. This event was recoded to loss of a child to death).

For analytic purposes, similar events were further combined such that seven distinct event types were considered: trauma during childhood, experiencing violence in adulthood, witnessing violence in adulthood, accidents, death of a child, war, and any other PTE. We also created a dichotomous variable to represent individuals who were and were not in the top quarter of number of PTEs reported. Individuals in the top quarter reported 6 or more PTEs.

#### Depressive symptoms

Depressive symptoms were assessed with the Patient Health Questionnaire-9 (PHQ-9) [22]. The PHQ-9 assesses depressive symptoms within the last two weeks. Scores of 10 or greater were categorized as moderate to severe depressive symptoms [22]. The PHQ-9 has been validated in French and with PWH in sub-Saharan Africa [23–26].

#### Anxiety symptoms

Anxiety symptoms were assessed with the General Anxiety Disorder-7 (GAD-7) [27]. The GAD-7 assesses anxiety symptoms within the past two weeks. Scores of 10 or greater were categorized as moderate or severe anxiety symptoms. The GAD-7 has been validated in French and among a population with a high prevalence of HIV in sub-Saharan Africa [28–31].

#### Post-traumatic stress disorder symptoms

Post-traumatic stress disorder (PTSD) symptoms were assessed with the PTSD Checklist for DSM-5 (PCL-5) [32]. The PCL-5 assesses the presence of PTSD symptoms in the past month. Scores of 31 or greater were categorized as symptoms of probable PTSD [33]. The PCL-5 has been validated in French and among a population with a high prevalence of HIV in Zimbabwe [34, 35].

#### Hazardous alcohol use

Hazardous alcohol use was assessed using the Alcohol Use Disorders Identification Test (AUDIT) which assesses the presence of hazardous or harmful drinking [36]. Scores of 8 or greater for men and 7 or greater for women were categorized as hazardous alcohol use.

#### Sociodemographic characteristics

Sociodemographic characteristics explored included age, gender, education, relationship status, employment, and number of children.

#### PTE and IPV

Individuals were asked about exposure to lifetime traumatic events including physical assault or abuse as an adult from an intimate partner and sexual assault or rape in adulthood, as described above. In addition, individuals also completed the Demographic and Health Survey (DHS) domestic violence module which captures four distinct domains of IPV: controlling behavior and emotional, sexual, and physical IPV [37]. This tool is a modified version of the Conflict Tactics Scale and asks respondents if they have experienced 15 separate behaviorally-specific types of IPV [38]. In our prior work, we noted discrepancies between reports of physical and sexual violence on the DHS domestic violence module and the modified version of the Life Events Checklist for DSM-5 used in the assessment of lifetime PTEs in this analysis [39]. Thus, we considered individuals who reported physical IPV with their most recent partner on the DHS domestic violence module, but did not report physical assault or abuse from a partner in adulthood on the PTE assessment to have experienced physical assault or abuse in adulthood from a partner in this analysis. Similarly, we considered individuals who reported sexual IPV with their most recent partner on the DHS domestic violence module but did not report sexual assault or rape as an adult on the PTE assessment to have experienced sexual assault or rape in adulthood.

### Missing data

A small number of individuals were missing data on individual items across the mental health scales (*n* = 6 for PHQ-9; *n* = 13 for PCL-5; *n* = 12 for GAD-7). For individuals missing data on less than 10% of items for a scale (*n* = 6 for PHQ-9; *n* = 13 for PCL-5; *n* = 10 for GAD-7), the mean of the individual’s non-missing scale responses was imputed for the missing items [40].

### Statistical analyses

We used counts, proportions, medians, and interquartile ranges (IQR) to describe the study population overall and the occurrence of PTEs among study participants by presence of mental health symptoms. Log binominal regression was used to estimate the association between each type of PTE and symptoms of each mental health disorder, separately. Adjusted analyses controlled for gender and clinic, the a priori covariates of interest. Clinic was included as a fixed effect variable as only three clinics were included in the study.

## Results

Among the 426 PWH included in this analysis, most were women (58.7%) and in a relationship (58.5%). Over 40% of participants were aged 40 or older (41.5%) and had completed secondary school or higher (41.5%) (Table 1). PTEs were commonly reported, with 96% of participants having experienced at least one PTE and 86% reporting more than one PTE. Overall, participants reported a median of 4 PTEs (IQR: 2–5). The PTEs most commonly reported were seeing someone seriously injured or killed (44.8%), seeing people hitting or harming one another in your family as a child (42.7%), physical assault or abuse from an intimate partner (41.8%) and witnessing physical assault or abuse (41.3%) (Table 2). Approximately one-quarter (27.0%) of participants reported having experienced a PTE other than the ones specified. Among those who indicated they experienced an ‘other’ type of PTE, half (51.3%) described the event as the death of a family member or friend. Across depression, PTSD, and anxiety, the median number of PTEs was significantly higher among participants with, compared to those without, symptoms of the disorder (Table 3).

**Table 1:** Sociodemographic characteristics of 426 people with HIV newly entering HIV care in Cameroon

	**Total** **(*n* = 426)** **N (%)**
Gender	
Male	176 (41.3)
Female	250 (58.7)
Age	
21–39	249 (58.5)
40 +	177 (41.5)
Education	
None	31 (7.3)
Primary	218 (51.2)
≥ Secondary	177 (41.5)
Relationship status	
Single	177 (41.5)
Partnered	249 (58.5)
Number of children	
0	79 (18.6)
≥ 1	345 (81.4)
Missing	2
Employment status	
Not working for pay	151 (35.4)
Working for pay	275 (64.6)

**Table 2:** Prevalence of reported lifetime potentially traumatic events among 426 adults entering HIV care in Cameroon

Event	Yes N(%)
**Traumatic events in childhood**	
Seeing people hitting or harming one another in your family as a child	182 (42.7)
Physical assault or abuse as child	108 (25.4)
Sexual assault or rape as child	34 (8.0)
**Violent traumatic events in adulthood**	
Physical assault or abuse in adult life from an intimate partner	178 (41.8)
Physical assault or abuse in adult life from someone other than an intimate partner	100 (23.5)
Sexual assault or rape as adult	111 (26.1)
**Witnessing violent traumatic events**	
Seeing someone physically assaulted or abused	176 (41.3)
Seeing someone seriously injured or killed	191 (44.8)
**Accident-related traumatic events**	
Natural disaster (e.g. hurricane, earthquake, flood)	63 (14.8)
Serious accident or fire at home or job	118 (27.7)
**Other traumatic events**	
War	146 (34.3)
Losing a child to death	147 (34.5)
Other traumatic event (individually specified)	115 (27.0)

**Table 3:** Reported lifetime potentially traumatic events (PTE) among 426 adults entering HIV care in Cameroon, stratified by mental health symptoms

	**Total**	**Depression**		**PTSD**		**Anxiety**^**a**^		**Hazardous Alcohol Use**	
	Overall (*n* = 426) N (%)	Yes (*n* = 87) N (%)	No (*n* = 339) N (%)	Yes (*n* = 67) N (%)	No (*n* = 359) N (%)	Yes (*n* = 83) N (%)	No (*n* = 341) N (%)	Yes (*n* = 166) N (%)	No (*n* = 260) N (%)
PTE during childhood									
Any	231 (54.2)	64 (27.7)	167 (72.3)	51 (22.1)	180 (77.9)	57 (24.8)	173 (75.2)	92 (39.8)	139 (60.2)
None	195 (45.8)	23 (11.8)	172 (88.2)	16 (8.2)	179 (91.8)	26 (13.4)	168 (86.6)	74 (37.9)	121 (62.0)
Violent PTE (adulthood)									
Any	251 (58.9)	62 (24.7)	189 (75.3)	50 (19.9)	201 (80.1)	58 (23.2)	192 (76.8)	100 (39.8)	151 (60.2)
None	175 (41.1)	25 (14.3)	150 (85.7)	17 (9.7)	158 (90.3)	25 (14.4)	149 (85.6)	66 (37.7)	109 (62.3)
PTE in childhood and violent PTE (adulthood)									
Any	164 (38.5)	50 (30.5)	114 (69.5)	43 (26.2)	121 (73.8)	47 (28.8)	116 (71.2)	65 (39.6)	99 (60.4)
None	262 (61.5)	37 (14.1)	225 (85.9)	24 (9.2)	238 (90.8)	36 (13.8)	225 (86.2)	101 (38.5)	161 (61.5)
Witnessing violent PTE (adulthood)									
Any	256 (60.1)	58 (22.7)	198 (77.3)	48 (18.8)	208 (81.2)	56 (22.0)	199 (78.0)	107 (41.8)	149 (58.2)
None	170 (39.9)	29 (17.1)	141 (82.9)	19 (11.2)	151 (88.8)	27 (16.0)	142 (84.0)	59 (34.7)	111 (65.3)
Accident related PTE									
Any	153 (35.9)	37 (24.2)	116 (75.8)	25 (16.3)	128 (83.7)	33 (21.7)	119 (78.3)	68 (44.4)	85 (55.6)
None	273 (64.1)	50 (18.3)	223 (81.7)	42 (15.4)	231 (84.6)	50 (18.4)	222 (81.6)	98 (35.9)	175 (64.1)
Death of a child									
Any	147 (34.5)	28 (19.0)	119 (81.0)	27 (18.4)	120 (81.6)	28 (19.2)	118 (80.8)	57 (38.8)	90 (61.2)
None	279 (65.5)	59 (21.1)	220 (78.9)	40 (14.3)	239 (85.7)	55 (19.8)	223 (80.2)	109 (39.1)	170 (60.9)
War									
Any	146 (34.3)	36 (24.7)	110 (75.3)	33 (22.6)	113 (77.4)	33 (22.6)	113 (77.4)	60 (41.1)	86 (58.9)
None	280 (65.7)	51 (18.2)	229 (81.8)	34 (12.1)	246 (87.9)	50 (18.0)	228 (82.0)	106 (37.9)	174 (62.1)
Other (specified)									
Any	115 (27.0)	26 (22.6)	89 (77.4)	22 (19.1)	93 (80.9)	23 (20.2)	91 (79.8)	52 (45.2)	63 (54.8)
None	311 (73.0)	61 (19.6)	250 (80.4)	45 (14.5)	266 (85.5)	60 (19.4)	250 (80.6)	114 (36.7)	197 (63.3)
PTE count (median/IQR)	4 (2–5)	5 (3–6)	3 (2–5)	5 (4–7)	3 (2–5)	5 (3–6)	3 (2–5)	4 (3–6)	4 (2–5)
Lower 75^th^	326 (76.5)	53 (16.3)	273 (83.7)	39 (12.0)	287 (88.0)	55 (16.9)	270 (83.1)	118 (36.2)	208 (63.8)
Upper 25^th^	100 (23.5)	34 (34.0)	66 (66.0)	28 (28.0)	72 (72.0)	28 (28.3)	71 (71.7)	48 (48.0)	52 (52.0)

*Abbreviations*: *PTE* potentially traumatic event, *PTSD* post-traumatic stress disorder, *IQR* interquartile range

^a^missing anxiety *n* = 2

In multivariable analyses adjusted for gender and clinic the prevalence of probable PTSD was significantly higher among those who reported experiencing PTEs during childhood (aPR 2.0 [95% CI 1.1, 3.3]), violent PTEs during adulthood (aPR 1.7 [95% CI 1.0, 2.7]), and the death of a child (aPR 1.6 [95% CI 1.1, 2.5]) (Table 4). The prevalence of probable PTSD was also significantly higher among those who reported both PTEs in childhood and violent PTEs in adulthood (aPR 2.0 [95% CI 1.2, 3.2]). In multivariable analyses, experiencing both PTEs in childhood and a violent PTE during adulthood was associated with greater prevalence of anxiety symptoms (aPR 1.6 [95% CI 1.1, 2.4]). None of the specified types of PTEs were positively associated with the prevalence of symptoms of depression or hazardous alcohol use in adjusted analyses. However, experiencing an ‘other’ type of PTE was associated with significantly greater prevalence of depression (aPR 1.5, [95% CI 1.1, 2.3]); PTSD (aPR 2.1 [95% CI 1.4, 3.3]); and hazardous alcohol use (aPR 1.3 [95% CI 1.0, 1.6])) after adjustment. Experiencing a total number of PTE types in the top quarter (i.e., 6 or more PTEs) compared to the bottom-three quarters (i.e., 0–5 PTEs) was associated with significantly greater prevalence of symptoms of PTSD (aPR 1.6 [95% CI 1.1, 2.5]) and hazardous alcohol use (aPR 1.4 [95% CI 1.1, 1.9]).

**Table 4:** Unadjusted and adjusted associations between lifetime potentially traumatic events (PTEs) and mental health symptoms, among 426 adults entering HIV care in Cameroon

	**Depression**		**PTSD**		**Anxiety**^**b**^		**Hazardous alcohol use**	
	uPR (95% CI)	aPR (95% CI)^a^	uPR (95% CI)	aPR (95% CI)^a^	uPR (95% CI)	aPR (95% CI)^a^	uPR (95% CI)	aPR (95% CI)^a^
PTE during childhood	2.35 (1.52, 3.63)	1.45 (0.95, 2.19)	2.69 (1.59, 4.56)	1.96 (1.15, 3.33)	1.85 (1.21, 2.82)	1.47 (0.96, 2.26)	1.05 (0.83, 1.33)	1.06 (0.84, 1.33)
Violent PTE (adulthood)	1.73 (1.13, 2.64)	1.34 (0.90, 1.98)	2.05 (1.22, 3.43)	1.66 (1.01, 2.73)	1.61 (1.05, 2.48)	1.37 (0.90, 2.07)	1.06 (0.83, 1.35)	1.13 (0.89, 1.43)
PTE in childhood and violent PTE (adulthood)	2.16 (1.48, 3.15)	1.34 (0.93, 1.92)	2.86 (1.81, 4.53)	1.99 (1.25, 3.17)	2.09 (1.42, 3.08)	1.62 (1.09, 2.41)	1.03 (0.81, 1.31)	1.06 (0.83, 1.35)
Witnessing violent PTE (adulthood)	1.33 (0.89, 1.98)	0.91 (0.62, 1.33)	1.68 (1.02, 2.75)	1.17 (0.71, 1.91)	1.37 (0.91, 2.08)	1.13 (0.75, 1.72)	1.20 (0.94, 1.55)	1.11 (0.86, 1.44)
Accident-related PTE	1.32 (0.91, 1.92)	1.38 (0.99, 1.93)	1.06 (0.67, 1.67)	1.15 (0.75, 1.77)	1.18 (0.80, 1.75)	1.35 (0.93, 1.95)	1.24 (0.98, 1.57)	1.13 (0.90, 1.42)
Death of a child	0.90 (0.60, 1.35)	1.12 (0.77, 1.61)	1.28 (0.82, 2.00)	1.63 (1.08, 2.46)	0.97 (0.64, 1.46)	1.17 (0.79, 1.73)	0.99 (0.77, 1.28)	1.07 (0.85, 1.36)
War	1.35 (0.93, 1.97)	0.67 (0.45, 0.99)	1.86 (1.20, 2.88)	0.89 (0.54, 1.49)	1.26 (0.85, 1.86)	0.66 (0.43, 1.01)	1.09 (0.85, 1.39)	1.02 (0.77, 1.37)
Other (specified)	1.15 (0.77, 1.73)	1.55 (1.06, 2.26)	1.32 (0.83, 2.10)	2.13 (1.38, 3.27)	1.04 (0.68, 1.60)	1.39 (0.90, 2.15)	1.23 (0.96, 1.58)	1.28 (1.02, 1.61)
*PTE Count*								
Upper 25^th^	2.09 (1.45, 3.02)	1.32 (0.93, 1.88)	2.34 (1.52, 3.60)	1.63 (1.07, 2.48)	1.67 (1.13, 2.48)	1.31 (0.89, 1.92)	1.33 (1.03, 1.70)	1.44 (1.10, 1.89)
Lower 75^th^	1.00	1.00	1.00	1.00	1.00	1.00	1.00	1.00

*Abbreviations*: *PTE* potentially traumatic event, *PTSD* post-traumatic stress disorder, *uPR* unadjusted prevalence ratio, *aPR* adjusted prevalence ratio, *CI* confidence interval

^a^adjusted for gender and clinic

^b^missing anxiety *n* = 2

## Discussion

Experiences of PTEs were extremely common among this group of PWH initiating HIV care in Cameroon. Overall, 96% of respondents reported at least one PTE and 86% reported more than one PTE. The median number of reported PTEs was four, with exposure to a greater number of PTEs associated with a significantly greater prevalence of all mental health disorders assessed. The most commonly reported PTEs were witnessing violence, injury, or death and experiencing physical IPV. PTEs during childhood were also commonly reported with over half (54%) reporting at least one childhood PTE. The prevalence of probable PTSD was significantly higher among those who reported experiencing PTEs during childhood, violent PTEs during adulthood, and the death of a child. The prevalence of anxiety symptoms was significantly higher among those who reported experiencing both a PTE in childhood and violent PTE during adulthood. However, none of the specified types of PTEs were positively associated with the prevalence of symptoms of depression or hazardous alcohol use after adjustment.

Estimates of PTE prevalence among PWH in SSA remain limited. A study of individuals seeking HIV testing in South Africa found that 62% experienced at least one PTE while a study of women in HIV care in South Africa found that 81% had experienced at least one PTE [3, 11]. A study of PWH in Tanzania found that PWH reported a mean of three lifetime PTEs [12, 41]. Beyond SSA, a study with PWH in the US found that 64% reported having experienced at least one PTE while a study with PWH in the southern US found that 72% of participants experienced two or more PTEs [42, 43].

Estimates of the prevalence of childhood PTEs among PWH in SSA are also limited. The prevalence of childhood sexual abuse reported in this study (8%) was similar to that reported among a sample of PWH in Tanzania (6%) and in a community cohort that included individuals with and without HIV (7%) in Tanzania [12]. The prevalence of childhood physical abuse reported in this study (25%) was similar to the prevalence of childhood physical abuse estimated in Zimbabwe [44], but substantially higher than that reported among PWH in Tanzania (2%), a community cohort of people with and without HIV in Tanzania (2%), and a community sample of people with and without HIV in South Africa (8%) [12, 45]. However, measures of childhood abuse and maltreatment varied meaningfully across studies making direct comparisons challenging.

In the current study, in multivariable analyses, reporting a childhood PTE was associated with significantly increased prevalence of probable PTSD, but not symptoms of depression, anxiety, or hazardous alcohol use. This is in contrast to a systematic review of the relationship between child maltreatment and mental health among PWH that found that having experienced child maltreatment increased risk of PTSD, depression, and substance use in adulthood [7]. However, only one study included in that review was conducted outside the United States or Canada. A study of women with HIV in Haiti found that childhood sexual abuse was associated with greater severity of alcohol use and anxiety [46]. It should be noted that exposure to childhood abuse has also been associated with greater HIV prevalence and greater HIV sexual risk behavior in adulthood, including having sex under the influence of alcohol or drugs, concurrent sexual partnerships, and inconsistent condom use [47, 48]. However, the majority of studies that have examined the relationship among abuse during childhood, HIV, and mental health in adulthood are cross-sectional in nature. Longitudinal research is needed to better understand the incidence, persistence, and severity of mental health disorders among PWH who have experienced abuse in childhood and causal pathways among abuse in childhood, HIV infection, and mental health disorders [7]. Given the high prevalence of childhood PTEs and the mental health impact of childhood PTEs into adulthood, strategies focused on primary prevention of childhood abuse and maltreatment are needed. Greater understanding of the long-term impact of childhood abuse and maltreatment on the mental health of PWH in SSA is needed.

Violent PTEs in adulthood were also common among this group of PWH in Cameroon. Over 40% of respondents reported physical assault by a partner and 26% reported sexual violence in adulthood. This is similar to a meta-analysis which found that 33% of PWH in sub-Saharan Africa reported having experienced IPV and to a study of pregnant women living with HIV in Cameroon in which 37% reported physical IPV and 31% reported sexual IPV [9, 10]. HIV-related stigma is often a direct or indirect cause of violence against PWH and has been consistently associated with poor mental health. The current study is unable to distinguish between HIV- and non-HIV-related violent PTEs. Greater understanding of the longitudinal pathways among HIV- and non-HIV-related violence, HIV-related stigma, and mental health is needed. Having experienced a violent PTE in adulthood was associated with increased prevalence of PTSD. A systematic review of psychological therapies for women who experienced IPV found evidence that such therapies reduced symptoms of depression and anxiety [49]. However, findings related to PTSD were equivocal [49]. Interventions that concurrently address IPV and mental health remain limited [50]. Given the high prevalence of IPV among women with HIV and the relationship between violent PTEs and PTSD, integrated interventions that address IPV and mental health disorders should be developed, implemented, and evaluated. The effectiveness of integrated IPV and mental health interventions compared to single-component (that focus on IPV or mental health only) interventions should be explored.

More than one-third (34%) of respondents reported lifetime exposure to war. It should be noted that this study was conducted during a period of substantial political unrest in Cameroon in which armed violence was common. Surprisingly, having experienced war was not positively associated with mental health outcomes assessed in multivariable models. This is in contrast to previous research that has consistently found conflict and war to be associated with increased risk of mental health disorders, both among individuals directly engaged in conflict and civilians [51–53]. Longitudinal research that examines the long-term mental health impact of ongoing political unrest and violence in Cameroon is warranted.

Over one-third (34%) of respondents reported having experienced the loss of a child. This is higher than has been reported among PWH in Tanzania (25%), among postpartum women in Ethiopia (26%) and among a community sample of women with and without HIV in Tanzania (14%) [12, 54]. The prevalence of child loss in the current study may reflect elevated HIV prevalence among participants’ children, barriers to HIV diagnosis and ART initiation among pregnant women with HIV, and barriers to early HIV diagnosis and treatment among respondents’ children with HIV. It has been estimated that over one-third (36%) of pregnant women with HIV and almost two-thirds (65%) of children aged 0–14 years with HIV in Cameroon are not on ART [55].

Having experienced an ‘other’ type of PTE was associated with increased prevalence of depression, PTSD, and hazardous alcohol use symptoms. Notably, over half of the ‘other’ types of PTEs reported were the death of a friend or family member. In our previous work which assessed the relationship between recent stressful life events and mental health in this group of individuals entering HIV care in Cameroon, we found that reporting the recent death of a close friend or family member was significantly associated with reporting symptoms of probable PTSD, but not depression or anxiety after adjustment for gender and clinic [56]. Additional research is needed to understand the relationships among grief, bereavement, and mental health among PWH. The development and evaluation of interventions to support PWH with HIV- and non-HIV-related loss are needed. Cognitive-behavioral interventions have been found to improve grief and mental health outcomes among orphaned children in SSA. Less is known about how to effectively address grief, bereavement, and adverse mental health outcomes among adults with HIV [57, 58]. Given the prevalence of the loss of children and other loved ones in this group of PWH and the relationship between such loss and poor mental health, such research should be prioritized.

Surprisingly, hazardous alcohol use was significantly associated only with having experienced an ‘other’ type of PTE and with a greater number of PTEs in multivariable models. This is in contrast to previous research that has found many PTEs to be associated with substance use, including in SSA [59, 60]. However, we are not aware of prior research on the relationship between PTEs and alcohol use among PWH in Cameroon. Greater understanding of the relationship between alcohol use and PTEs among PWH in Cameroon and throughout SSA is warranted.

Given the high prevalence of PTEs, providers who care for PWH should provide a trauma-informed approach to care and adhere to trauma-informed practice recommendations. These include creating a trauma-informed health care environment to promote feelings of safety during the health care experience; routine, evidence-based trauma screening; patient psychoeducation about the relationship between trauma and HIV care and treatment; and referral to evidence-based mental health care, as needed. Screening for trauma and integrating trauma-focused interventions into HIV care settings should be prioritized. While limited, evidence suggests high acceptability of trauma screening among PWH, which may be related to the high prevalence of PTEs. A study of women with HIV in South Africa found that almost all (98%) thought that screening of traumatic experiences should be routinely integrated into HIV care [11]. Importantly, evidence suggests that significant variability in traumatic event identification exists across trauma screening tools. The prevalence of reported PTEs is greater when behaviorally specific, multiple-item traumatic event assessments are used as compared to single-item assessments or assessments that include items that are not behaviorally-specific [39, 61]. To most effectively assess PTEs, behaviorally-specific, multiple-item trauma screening tools should be used. Similar to many settings in SSA, mental health screening and treatment remains limited in Cameroon [19]. A study of PWH in Cameroon found that while 21% of participants met criteria for major depressive disorder just 12% had ever received depression treatment that was helpful or effective [62]. There is a clear and substantial unmet need for evidence-based mental health care for PWH in Cameroon. Such care should include trauma screening and referral to care as needed.

Most trauma-focused mental health interventions have been developed and evaluated in the United States and other high-resource settings. The efficacy of trauma-focused mental health interventions among PWH in SSA warrants urgent attention [63]. Studies to test the efficacy and effectiveness of such interventions should incorporate evaluation of implementation strategies to speed scale-up and integration of efficacious interventions into HIV care settings [63]. One intervention specifically developed to address trauma and mental health among women living with HIV in South Africa was associated with reductions in PTSD symptoms up to six months after the intervention [64]. While limited, evidence suggests that interventions focused on enhancing resilience may improve the mental health and well-being of PWH. A study with youth with HIV in Tanzania found that a mental health intervention that promoted resilience was associated with improved coping skills, reduced stigma, and improved relationships [65]. Beyond SSA, resilience-focused interventions with PWH in China were associated with decreased depression and anxiety and increased resilience and quality of life [66, 67]. A pilot study of a resilience-focused intervention with PWH in the United States found high feasibility and acceptability [68]. Mental health interventions that include resilience building components should be developed, implemented, and evaluated among PWH in SSA. Few trauma-focused mental health interventions have been developed to address trauma among men, transgender, or nonbinary individuals with HIV in SSA or other resource-limited settings [69]. In addition, little is known about whether trauma-focused mental health interventions are associated with improved HIV treatment outcomes. Greater understanding of the impact of trauma-focused mental health interventions on HIV care outcomes is needed, particularly as evidence suggests that mental health symptoms may mediate the relationship between trauma exposure and HIV treatment outcomes [12].

Longitudinal research is also needed to better understand potential mediators and moderators of the relationship between PTEs and mental health. While limited, research suggests that resilience may moderate the relationship between trauma and mental health symptoms. A study with women living with HIV in South Africa who had experienced trauma in childhood found greater resilience to be associated with lower likelihood of depression symptoms in adulthood [1]. Avoidant coping has also been shown to be associated with greater PTSD symptomology among PWH who have experienced trauma [70].

PTEs were commonly reported across the life course. Overall, respondents reported a mean of 4 distinct types of PTEs, and those reporting a greater number of PTE types had increased prevalence of all mental health disorders assessed. Many respondents (38%) reported both PTEs in childhood (i.e., seeing people hitting or harming one another in their family as a child, physical assault or abuse as a child, or sexual assault/rape as a child) and a violent PTE during adulthood (i.e., physical assault or abuse in adult life from partner, physical assault or abuse in adult life from someone other than partner, or sexual assault/rape as an adult). Research suggests that experiencing trauma in childhood and adulthood is associated with greater dissociative symptoms. The cumulative mental health impact of repeated exposure to PTE throughout the life course among PWH should be further explored.

This study has limitations worth noting. The study was conducted at three urban HIV treatment centers in Cameroon and may not be fully generalizable to other settings or populations. Further, while similar to commonly used trauma assessments, the trauma assessment tool used in the study has not been validated in Cameroon. In addition, the timing of reported PTEs is unknown, both in relation to mental health symptom onset and HIV diagnosis. Further, several potential confounders of the relationship between trauma and mental health, including socioeconomic status throughout the life course and parental mental health, were not measured in the current study. Thus, effect estimates may be influenced by uncontrolled confounding. Finally, because this study only included PWH, the extent to which the prevalence of traumatic events is similar or dissimilar to Cameroonian individuals without HIV or the general population in Cameroon remains unclear.

PTEs were very commonly reported among this group of PWH initiating HIV care in Cameroon and associated with mental health symptoms. Research is needed to foster the primary prevention of PTEs throughout the life course in PWH and to address the mental health sequelae of PTEs among PWH.

## Data Availability

Data used in this analysis are not publicly available at the present time. Data may be made available by author AMP on reasonable request.
